# The anthelmintic drug praziquantel activates a schistosome transient receptor potential channel

**DOI:** 10.1074/jbc.AC119.011093

**Published:** 2019-10-25

**Authors:** Sang-Kyu Park, Gihan S. Gunaratne, Evgeny G. Chulkov, Francie Moehring, Paul McCusker, Peter I. Dosa, John D. Chan, Cheryl L. Stucky, Jonathan S. Marchant

**Affiliations:** ‡ Department of Cell Biology, Neurobiology and Anatomy, Medical College of Wisconsin, Milwaukee, Wisconsin 53226; § Institute for Therapeutics Discovery and Development, University of Minnesota, Minneapolis, Minnesota 55414

**Keywords:** transient receptor potential channels (TRP channels), calcium channel, calcium imaging, infectious disease, ion channel, bilharzia, flatworm, schistosomiasis, Ca2+ signaling, parasite

## Abstract

The anthelmintic drug praziquantel (PZQ) is used to treat schistosomiasis, a neglected tropical disease that affects over 200 million people worldwide. PZQ causes Ca^2+^ influx and spastic paralysis of adult worms and rapid vacuolization of the worm surface. However, the mechanism of action of PZQ remains unknown even after 40 years of clinical use. Here, we demonstrate that PZQ activates a schistosome transient receptor potential (TRP) channel, christened *Sm*.TRPM_PZQ_, present in parasitic schistosomes and other PZQ-sensitive parasites. Several properties of *Sm*.TRPM_PZQ_ were consistent with known effects of PZQ on schistosomes, including (i) nanomolar sensitivity to PZQ; (ii) stereoselectivity toward (*R*)-PZQ; (iii) mediation of sustained Ca^2+^ signals in response to PZQ; and (iv) a pharmacological profile that mirrors the well-known effects of PZQ on muscle contraction and tegumental disruption. We anticipate that these findings will spur development of novel therapeutic interventions to manage schistosome infections and broader interest in PZQ, which is finally unmasked as a potent flatworm TRP channel activator.

## Introduction

Schistosomiasis (bilharzia) is a parasitic worm infection that infects millions of people worldwide (1, 2). Mature blood flukes living in the vasculature lay eggs, which become deposited in host tissues, where they trigger local inflammatory responses. Chronic infections become associated with fibrosis and obstructive disease in gastrointestinal tissues and liver (*Schistosoma mansoni*, *Schistosoma japonicum*), genitourinary disease (*Schistosoma haematobium*), anemia, undernutrition, and a heightened risk for other comorbidities (3). The annual disease burden has been estimated as a loss of up to 70 million disability-adjusted life years (1, 2).

In 2017, ∼100 million people (∼80 million school-aged children) received free preventive treatment for schistosomiasis. This treatment depends on a drug called praziquantel (PZQ),^2^ as no effective vaccine currently exists (4). The clinical formulation of PZQ is a racemate (±PZQ) composed of the enantiomers (*R*)-PZQ and (*S*)-PZQ. (*R*)-PZQ is the antischistosomal eutomer, known to cause Ca^2+^ influx and spastic paralysis of adult worms and rapid vacuolization of the worm tegumental surface (5). (*S*)-PZQ is regarded as the less active distomer (6). From a therapeutic perspective, it is problematic that despite decades of clinical usage, as well as demonstration of strains with lower sensitivity to PZQ in both laboratory and field, the flatworm target(s) of PZQ remains unknown (7, 8). This lack of knowledge is a longstanding roadblock for this field.

Here, we demonstrate that (*R*)-PZQ activates a Ca^2+^-permeable transient receptor potential (TRP) channel expressed in PZQ-sensitive flatworms.

## Results

**Figure 1.**
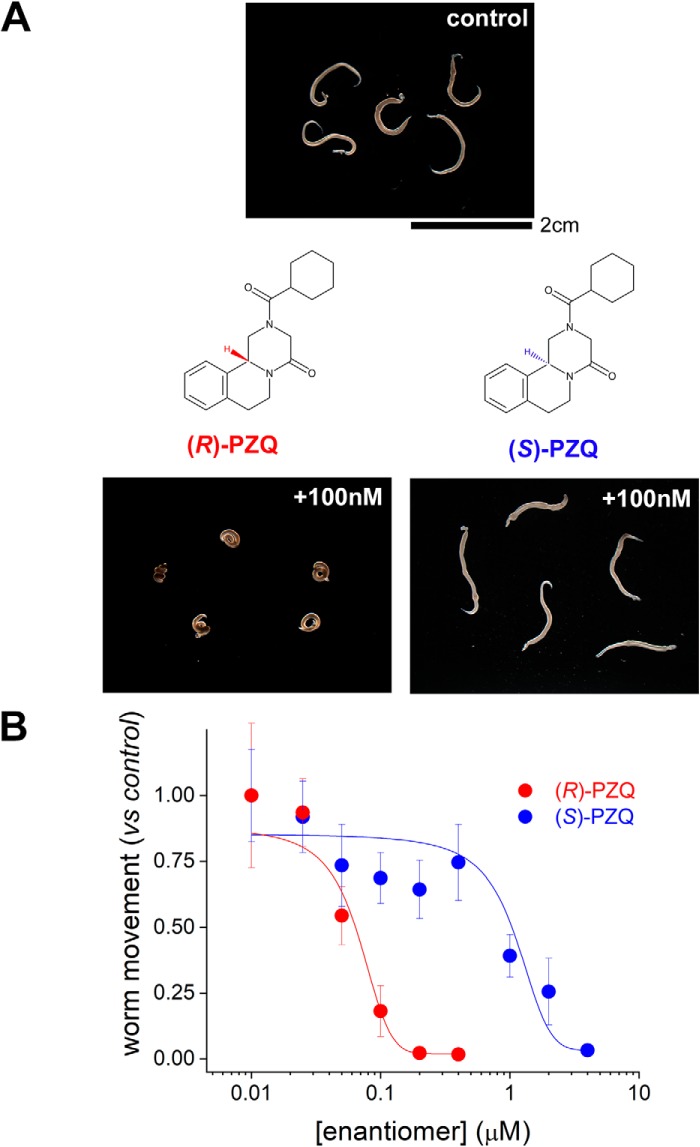
**Effects of PZQ enantiomers on schistosome worms *in vitro*.** *A*, images of five schistosome worms before (*top*) and 1 min after (*bottom*) the addition of a fixed concentration (100 nm) of (*R*)-PZQ or (*S*)-PZQ. Structures of (*R*)-PZQ and (*S*)-PZQ, tetracyclic tetrahydroisoquinolines, highlight chirality. *B*, concentration–response relationships for (*R*)-PZQ (*red*) and (*S*)-PZQ (*blue*) evoked changes in worm motility measured as described (13). Data represent mean ± S.D. (*error bars*) for at least three independent experiments.

The addition of (*R*)-PZQ (100 nm) to adult schistosome worms *ex vivo* caused a rapid, spastic paralysis (Fig. 1*A*). The addition of the same concentration of (*S*)-PZQ was ineffective at causing contraction (Fig. 1*A*). This demonstrates the differential potency of the two PZQ enantiomers against adult schistosome worms (EC_50_ for (*R*)-PZQ = 68 ± 7 nm, EC_50_ for (*S*)-PZQ = 1.1 ± 0.4 μm; Fig. 1*B*) observed both *ex vivo* and *in vivo* (6).

Although no binding site(s) for these enantiomers has been identified in parasitic flatworms, there has been considerable recent progress in identifying targets for (*R*)-PZQ and (*S*)-PZQ in the human host (9). (*R*)-PZQ is a partial agonist of the human 5-hydroxytryptamine 2B receptor (5HT_2B_R (10)), and (*S*)-PZQ is a partial agonist of the human transient receptor potential melastatin-8 channel (hTRPM8 (11)). Whereas regulation of these host targets occurs over the micromolar range (10–12), molecular divergence between human and flatworm ligand-binding pockets (13, 14) makes it reasonable to anticipate different binding poises and affinities at a homologous schistosome target(s).

**Figure 2.**
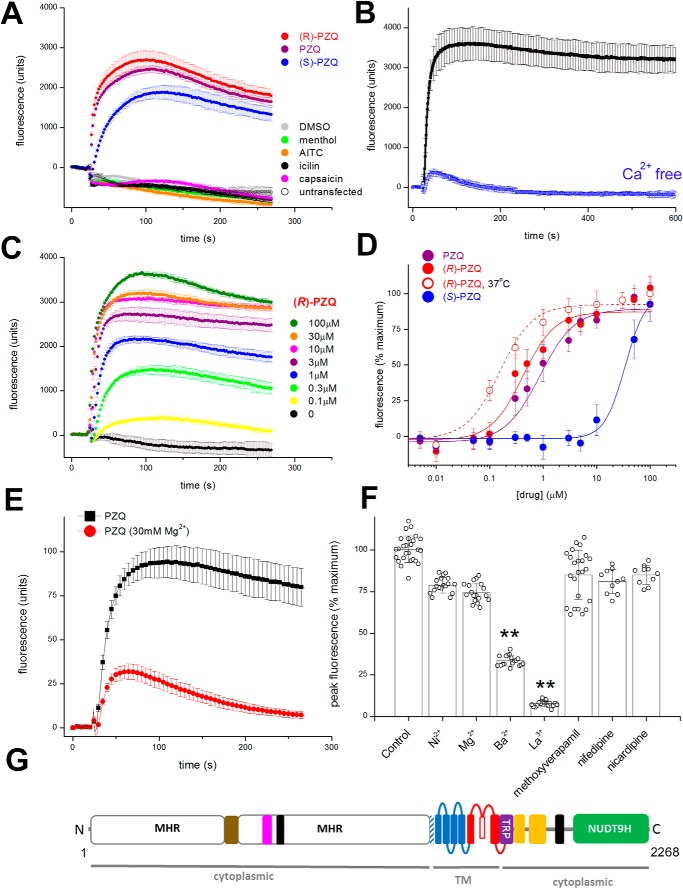
**Properties of *Sm*.TRPM_PZQ_.** *A*, Fluo-4 fluorescence traces in HEK293 cells in expressing *Sm*.TRPM_PZQ_ monitored prior to and after the addition of ±PZQ (*purple*), (*R*)-PZQ (*red*), and (*S*)-PZQ (*blue*) (50 μm). Other tested TRP ligands (menthol (*green*), AITC (*orange*), icilin (*black*), and capsaicin (*magenta*) (50 μm)) did not evoke a response. For Fig. 2, all Ca^2+^ reporter assays were performed using a FLIPR to resolve Fluo-4 fluorescence from cells in 96-well plates. Data are presented as mean ± S.D. (*error bars*) of technical replicates in an individual experiment except where stated. *B*, PZQ-evoked Ca^2+^ signals depend on Ca^2+^ influx. Responses to ±PZQ (5 μm) in HEK293 cells expressing *Sm*.TRPM_PZQ_ in normal HBSS (*black*) and Ca^2+^-free HBSS (*blue*, HBSS supplemented with 1 mm EGTA). *C*, fluorescence traces showing action of various concentrations (100 nm to 100 μm) of (*R*)-PZQ at *Sm*.TRPM_PZQ_. *D*, concentration–response relationships for ±PZQ (*purple*), (*R*)-PZQ (*red*), and (*S*)-PZQ (*blue*) at *Sm*.TRPM_PZQ_ from experiments performed at room temperature. An (*R*)-PZQ concentration–response curve performed at 37 °C is shown by the *dashed red line*. Data are mean ± S.D. for at least three independent experiments. *E*, effect of Mg^2+^ concentration on *Sm*.TRPM_PZQ_ activity. Assays were performed under the same medium conditions as described previously (15), comparing the effects of 1 μm ±PZQ at 0.4 mm Mg^2+^, 0.4 mm Ca^2+^ (1:1) and 30 mm Mg^2+^, 0.4 mm Ca^2+^ (75:1). *F*, pharmacological signature of *Sm*.TRPM_PZQ_. Effects of various heavy metal ions (10 mm) on ±PZQ (1 μm) evoked *Sm*.TRPM_PZQ_ activity to replicate conditions reported previously (17). Effects of drugs were as follows: methoxyverapamil (D-600, 100 μm; mimicking conditions in Ref. 17) and nifedipine and nicardipine (20 μm; mimicking conditions in Ref. 18). Data show individual data points (mean ± S.D.) from a total of at least three independent transfections. One-way analysis of variance yielded significant variance between treatments. An ensuing post hoc Tukey test showed that both the Ba^2+^ and La^3+^ treatments differed significantly from other conditions (**, *p* < 0.01). *G*, domain organization of *Sm*.TRPM_PZQ_. The *schematic* shows distinct domains identified in recent TRPM2 structures to include the N-terminal TRPM homology region (MHR) domain containing an ankyrin-like repeat domain (*brown* (22)), the pre-S1 helix (*shaded*), the six TM-spanning helices (*S1–S6*) comprising the voltage sensor–like domain (*VSLD*; *blue*) and pore-forming domain (*red*), the TRP domain (*purple*), the rib and pole helices (*yellow*), an additional helical domain (*black*), and the C-terminal NUDT9H domain.

Following this logic, we searched for flatworm TRP channels exhibiting sequence homology to hTRPM8. One candidate, christened *Sm*.TRPM_PZQ_, mediated robust Ca^2+^ signals in response to ±PZQ and (*R*)-PZQ in transfected HEK293 cells that were not observed in either untransfected or vehicle-treated cells expressing *Sm*.TRPM_PZQ_ (Fig. 2*A*). (*S*)-PZQ also evoked a response in *Sm*.TRPM_PZQ_-expressing cells, but with slower kinetics suggestive of a stereoselectivity toward the PZQ enantiomers that would be poorly discriminated at the high concentration of the primary screening (50 μm; Fig. 2*A*). Established mammalian TRP ligands (menthol, allyl isothiocyanate (AITC), icilin, and capsaicin) did not activate *Sm*.TRPM_PZQ_ (Fig. 2*A*). The PZQ-evoked Ca^2+^ signal depended on Ca^2+^ entry across the plasma membrane, as removal of extracellular Ca^2+^ abolished the sustained cytoplasmic Ca^2+^ elevation (Fig. 2*B*).

Full concentration–response curves were performed with (*R*)-PZQ (Fig. 2, *C* and *D*), (*S*)-PZQ, and ±PZQ (Fig. 2*D*). *Sm*.TRPM_PZQ_ was activated by ±PZQ (EC_50_ = 1.08 ± 0.14 μm; Fig. 2*D*), and activation was stereoselective, with (*R*)-PZQ evoking Ca^2+^ signals over a considerably lower concentration range (EC_50_ = 597 ± 10 nm) than (*S*)-PZQ (EC_50_ = 27.9 ± 3.1 μm; Fig. 2*D*). When the incubation temperature was increased to 37 °C, (*R*)-PZQ activated *Sm*.TRPM_PZQ_ over an even lower concentration range (EC_50_ = 154 ± 33 nm; Fig. 2*D*).

Early work on schistosomes established key pharmacological characteristics of PZQ action on parasite muscle contraction and/or ^45^Ca^2+^ uptake. These include (i) conversion of contraction from sustained to phasic in the presence of elevated Mg^2+^, (ii) inhibition by La^3+^, and (iii) insensitivity to several voltage-operated Ca^2+^ channel (Ca_v_) blockers at specific doses. We therefore examined the impact of these same manipulations on *Sm*.TRPM_PZQ_ activity. First, increasing the Mg^2+^/Ca^2+^ ratio to a level (75:1) that resulted in transient muscle contraction (15, 16) also resulted in a transient PZQ-evoked Ca^2+^ signal via *Sm*.TRPM_PZQ_ (Fig. 2*E*). Second, preincubation of worms with La^3+^ (10 mm) inhibited both PZQ-evoked ^45^Ca^2+^ accumulation and PZQ-evoked contraction (17). La^3+^ (10 mm) also inhibited *Sm*.TRPM_PZQ_ activity (Fig. 2*F*). Third, three Ca_v_ blockers (methoxyverapamil, nifedipine, and nicardipine) that failed to block PZQ action on worms (17, 18) also failed to inhibit PZQ-evoked *Sm*.TRPM_PZQ_ activity at the same doses (Fig. 2*F*). Therefore, the pharmacological properties of *Sm*.TRPM_PZQ_ mirror the characteristics of PZQ action on schistosome muscle.

Consistent with the homology-based search strategy, *Sm*.TRPM_PZQ_ is a member of the TRP melastatin (TRPM) subfamily. Sequence analysis revealed an architecture characteristic of TRPM channels (Fig. 2*G*), a well-represented family within flatworm genomes (19). Features include a long N-terminal TRPM homology region (MHR) domain, followed by six predicted transmembrane (TM) domains with a pore-forming re-entry loop between TM5 and TM6, a conserved TRP helix juxtaposed to coiled-coil regions, and a cytoplasmic C-terminal enzymatic domain (Fig. 2*G*). This enzyme domain displayed homology with the human ADP-ribose (ADPR) pyrophosphatase NUDT9, a feature characteristic of TRPM2 channels (20–23). TRPM2 and TRPM8 are closely related “long” TRPM channels, and *Sm*.TRPM_PZQ_ displays the highest sequence identity with these human TRPM variants (29.5 and 28.5% sequence identity with hTRPM2 and hTRPM8, respectively).

Analysis of flatworm genomic and transcriptomic data sets revealed the presence of *Sm*.TRPM_PZQ_ homologs in other parasitic flatworms, including cestodes and flukes, known to exhibit PZQ sensitivity (Fig. S1*A*). To assess the broader PZQ sensitivity of schistosome TRP channels, we screened three other TRPs. First, we examined the previously characterized *Sm*.TRPA, which has been shown to activated by the ligands AITC and capsaicin (14). *Sm*.TRPA did not respond to PZQ but, as expected, did respond to the other two compounds (Fig. S1*B*). Next, we focused on the schistosome TRPM subfamily, which is predicted to contain seven members (Fig. S1*A*). The two members most closely related to *Sm*.TRPM_PZQ_ (Smp_130890 and Smp_000050) did not respond to PZQ (Fig. S1, *C* and *D*). With the caveat that there is no control for functional expression, as endogenous agonists of these TRPM channels are unknown, these data suggest that schistosome TRP (and TRPM) channels are not broadly sensitive to PZQ.

**Figure 3.**
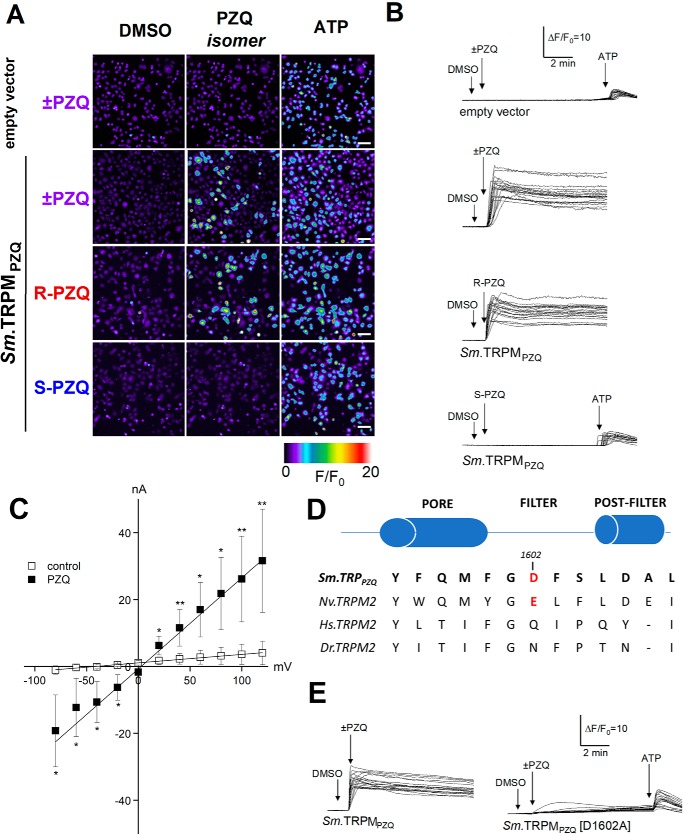
**Characterization of *Sm*.TRPM_PZQ_-mediated Ca^2+^ signals by confocal imaging and electrophysiology.** *A*, confocal Ca^2+^ imaging of Fluo-4 fluorescence from control (*top*) or *Sm*.TRPM_PZQ_-expressing HEK cells, stimulated with ±PZQ, individual PZQ enantiomers (1 μm), or ATP (100 μm). *Pseudocolored images* of the imaging field are captured at the indicated time points on related fluorescence traces shown in *B. Scale bar*, 40 μm. *B*, representative fluorescence traces from HEK293 cells loaded with Fluo-4 AM following the addition of DMSO vehicle, PZQ (10 μm for empty vector control transfections, 1 μm in Sm.TRPM_PZQ_-expressing cells), and ATP (100 μm). Time courses of fluorescence ratios are presented as Δ*F*/*F*_0_ (where Δ*F* represents change in fluorescence from baseline and *F*_0_ represents fluorescence at time 0). *C*, dependence of transmembrane current of *Sm.*TRPM_PZQ_-expressing HEK293 cells in whole-cell mode on the voltage in the absence (control, *open squares*) or presence of 2 μm PZQ (*closed squares*) in the bathing solution. Statistical analysis between *I*-V curve values in drug-treated and control conditions was performed using a Mann–Whitney test at each voltage condition (mean ± S.D. (*error bars*); *, *p* < 0.1; **, *p* < 0.05). Average membrane conductance and reversal potential in the absence and the presence of PZQ were 26 and 257 nS (*p* = 0.026) and −32 and −14 mV (*p* = 0.074), respectively. Data show peak current values from cells recorded from *n* ≥ 3 independent transfections. *D*, sequence alignment of the channel selectivity filter region in various TRPM2 channels to highlight the Asp-1602 residue in *Sm*.TRP_PZQ_ aligned with corresponding amino acid sequence from *N. vectensis* (*Nv.TRPM2*), human TRPM2 (*Hs.TRPM2*), and zebrafish TRPM2 (*Dr.TRPM2*). *E*, Ca^2+^-imaging data showing responses of control HEK cells (*top*), HEK cells expressing WT *Sm*.TRPM_PZQ_ (*middle*), and D1602A mutant (*bottom*) to ±PZQ (10 μm for empty vector control transfection, 1 μm in Sm.TRPM_PZQ_- or Sm.TRPM_PZQ_[D1602A]-expressing cells) from one of three representative experiments.

Next, to resolve the single-cell kinetics of *Sm*.TRPM_PZQ_ activity, we performed confocal Ca^2+^ imaging. In HEK cells transfected with empty vector, the addition of ±PZQ (10 μm) failed to evoke a cytoplasmic Ca^2+^ signal (Fig. 3, *A* and *B*), although cells responded to ATP (100 μm), which activated endogenous purinoceptors. In contrast, in HEK cells transiently transfected with *Sm*.TRPM_PZQ_, the addition of ±PZQ (1 μm) evoked a rapid and protracted rise in cytoplasmic Ca^2+^ (Fig. 3, *A* and *B*). Responses were evoked by (*R*)-PZQ, with (*S*)-PZQ being ineffective at the same concentration (1 μm; Fig. 3, *A* and *B*). The large and persistent increase in fluorescence evidenced little *Sm*.TRPM_PZQ_ desensitization in the presence of ±PZQ and contrasted with the smaller, transient nature of Ca^2+^ signals evoked by ATP. This signal was triggered by Ca^2+^ influx, as this response was seen only when Ca^2+^-containing medium was re-added to HEK cells initially exposed to ±PZQ in Ca^2+^-free medium (Fig. S2*A*). Activation of *Sm*.TRPM_PZQ_ by ±PZQ was also reversible, as ±PZQ washout resulted in a decrease of signal to baseline (Fig. S2*B*).

Electrophysiological analysis of *Sm*.TRPM_PZQ_ was performed by measuring whole-cell currents in HEK cells expressing GFP alone or expressing GFP and *Sm*.TRPM_PZQ_. In cells expressing GFP alone, the addition of ±PZQ (2 μm) did not evoke currents (0 of 18 cells examined). In contrast, in HEK cells co-transfected with cDNA encoding both *Sm*.TRPM_PZQ_ and GFP, the addition of ±PZQ evoked rapidly activating inward currents in all GFP-positive cells (22 of 22 cells, holding potential of −40 mV). Characterization of current magnitude after various voltage steps, in the absence and presence of PZQ (2 μm), revealed PZQ-activated *Sm*.TRPM_PZQ_-conducted large inward and outward currents with a linear *I*-V relationship (Fig. 3*C*), resembling the linear *I*-V relationship displayed by hTRPM2 channels (24). Based on sequence homology with another invertebrate TRPM2 channel (*Nematostella vectensis* TRPM2, *Nv*.TRPM2) that has been structurally and functionally characterized (25), we speculated that the substantial Ca^2+^ permeability of *Sm*.TRPM_PZQ_ (Fig. 3, *B* and *C*) is supported by the presence of a negatively charged residue in the predicted pore filter of *Sm*.TRPM_PZQ_ (*FGD* in Fig. 3*D*). This closely resembles the pore filter sequence of *Nv*.TRPM2 (*YGE* in Fig. 3*D*), which displays substantial Ca^2+^ permeability (25). Consistent with this idea, PZQ-evoked Ca^2+^ signals were strongly attenuated in HEK cells expressing the mutant *Sm*.TRPM_PZQ_[D1602A] (Fig. 3*E*). *Sm*.TRPM_PZQ_ therefore displays several characteristics consistent with the properties of TRPM2 channels.

## Discussion

These data represent the first report of a flatworm target activated by PZQ. Although further experiments would be needed to confirm *Sm*.TRPM_PZQ_ as *the* clinically relevant target in worms, our data clearly evidence *Sm*.TRPM_PZQ_ as *a* schistosome target of PZQ.

The properties of *Sm*.TRPM_PZQ_, a TRPM2-like channel, are, however, consistent with several key facets of PZQ action on worms. These include (i) nanomolar sensitivity to PZQ (Fig. 2, *C* and *D*); (ii) stereoselectivity toward (*R*)-PZQ (Figs. 2 and 3); (iii) mediation of a sustained Ca^2+^ entry in response to PZQ (Fig. 3*B*) that parallels the kinetics of worm contracture and tegumental disruption (15–17, 26); (iv) partial blockade by Mg^2+^ and complete inhibition by La^3+^, mirroring the effects of PZQ on muscle contraction and tegumental disruption (15–17, 26); (v) insensitivity to specific Ca_v_ blockers that fail to block PZQ action on worms (Fig. 2*F*) (16–18); and (vi) presence of homologs in other parasitic flatworms sensitive to PZQ (Fig. S1). Just as *Sm*.TRPM_PZQ_ supports long-lasting cellular Ca^2+^ signals (Figs. 2 and 3), human TRPM2 (hTRPM2) also exhibits long channel opening times that support substantial Ca^2+^ influx (23, 27). hTRMP2 is a well-known effector of apoptosis being responsive to reactive oxygen species through activation by H_2_O_2_ and ADPR (23, 28). Activation of hTRMP2 at the cell surface and within intracellular organelles causes lysosomal permeabilization and cell death (28–30). Such regulation could underpin the deleterious actions of ±PZQ on worm tegument crucial for the *in vivo* efficacy of PZQ (31, 32). Therefore, there are many similarities between the properties of *Sm*.TRPM_PZQ_ and the characteristics of PZQ action on schistosomes.

This discovery also prompts new questions. What are the endogenous agonists and/or environmental cues that regulate *Sm*.TRPM_PZQ_ activity across the parasite life cycle? In what cell type(s) is *Sm*.TRPM_PZQ_ expressed? How is *Sm*.TRPM_PZQ_ activity regulated in juvenile worms known to be less sensitive to PZQ? Is *Sm*.TRPM_PZQ_ activity altered in schistosome strains that show refractoriness to PZQ action? Mutagenesis demonstrates that single amino acid changes in *Sm*.TRPM_PZQ_ can dramatically alter channel responses to ±PZQ (Fig. 3*E*). This discovery also prioritizes analyses of TRPM_PZQ_ homologs in other flatworms as well as all other schistosome TRPM channels to assess broader PZQ sensitivity.

Finally, we note that (*R*)-PZQ is a potent activator of *Sm*.TRPM_PZQ_ (Fig. 2). Known regulators of hTRPM2, including the endogenous agonist ADPR (23), act over the micromolar range. This is important as hTRPM2 is an emerging clinical target for several nervous system and inflammatory disorders (23, 27). Understanding the basis of (*R*)-PZQ affinity for *Sm*.TRPM_PZQ_ and comparing regulation and gating of *Sm*.TRPM_PZQ_ with recently solved TRPM structures (20–22, 33) may reciprocally catalyze drug design at this clinically important human target.

## Experimental procedures

### Reagents

Enantiomers of ±PZQ were resolved following the protocol of Woelfle *et al.* (34). All chemical reagents were from Sigma. Cell culture reagents were from Invitrogen. Lipofectamine 2000 was from Thermo Fisher Scientific.

### Adult schistosome mobility assays

Adult schistosomes were recovered by dissection of the mesenteric vasculature in female Swiss Webster mice previously infected (∼49 days) with *S. mansoni* cercariae (NMRI strain) by the Schistosomiasis Resource Center at the Biomedical Research Institute (Rockville, MD). All animal experiments followed ethical regulations approved by the Medical College of Wisconsin institutional animal care and use committee. Harvested schistosomes were washed in RPMI 1640 supplemented with HEPES (25 mm), 5% heat-inactivated fetal bovine serum (FBS) (Gibco), and penicillin-streptomycin (100 units/ml) and incubated overnight (37 °C/5% CO_2_) in vented Petri dishes (100 × 25 mm). The following day, movement assays were performed using male worms in 6-well dishes (∼5 individual worms/3 ml of medium per well). Video recordings were captured using a Zeiss Discovery v20 stereomicroscope with a QiCAM 12-bit cooled color CCD camera controlled by Metamorph imaging software. Recordings (1 min) of worm motility (4 frames/s), during the addition of various drug concentrations were analyzed as described previously (13).

### Molecular cloning

For cloning of *Sm*.TRPM_PZQ_, total RNA was isolated from adult schistosome worm pairs using TRIzol® and poly(A)-purified using a NucleoTrap® mRNA minikit. cDNA was synthesized using the SuperScript^TM^ III first-strand synthesis system (Invitrogen). Using the predicted sequence (Smp_246790) as a template, cDNA from transcribed sequences was amplified by PCR (LA Taq^TM^ polymerase) and ligated into pGEM®-T Easy (Promega) for sequencing. Several splice variants of *Sm*.TRPM_PZQ_ were identified within both the N-terminal TRPM homology region (MHR) and cytoplasmic C-terminal domain, which will be characterized elsewhere. The sequence used here for functional analyses represents the reference sequence (2268 amino acids, Smp_246790.5).

### Cell culture and transfection

HEK293 cells (ATCC CRL-1573.3) and U2OS cells (ATCC HTB-96; Fig. S2*A* and *B*) were cultured in Dulbecco's modified Eagle's medium supplemented with 10% FBS, penicillin (100 units/ml), streptomycin (100 μg/ml), and l-glutamine (290 μg/ml). For screening parasite TRP channels, codon-optimized cDNAs and mutants (Genscript) were transiently transfected into HEK293 cells using Lipofectamine-2000 at a density of 3 × 10^6^ cells/dish (100 mm).

### Ca^2+^-imaging assays

Ca^2+^-imaging assays were performed using a fluorescence imaging plate reader (FLIPR^TETRA^, Molecular Devices). HEK293 cells (naive or transfected) were seeded (50,000 cells/well) in a black-walled clear-bottomed poly-d-lysine–coated 96-well plate (Corning) in Dulbecco's modified Eagle's medium supplemented with 10% dialyzed FBS. After 24 h, growth medium was removed, and cells were loaded with a fluorescent Ca^2+^ indicator (Fluo-4 direct dye, Invitrogen) by incubation (100 μl per well, 1 h at 37 °C) in Hanks' balanced salt solution (HBSS) assay buffer containing probenecid (2.5 mm) and HEPES (20 mm). Drug dilutions were prepared in assay buffer, without probenecid and dye, in V-shaped 96-well plates (Greiner Bio-one, Frickenhausen, Germany). After loading, the Ca^2+^ assay was performed at room temperature. Basal fluorescence was monitored for 20 s, and then 25 μl of each drug was added, and the signal (raw fluorescence units) was monitored over an additional 250 s. For quantitative analyses, peak fluorescence in each well was normalized to maximum -fold increase over baseline.

For confocal Ca^2+^ imaging, HEK cells were loaded with Fluo-4-AM (4 μm) and Pluronic F127 (0.4%) for 25 min at room temperature. Cells were then washed twice with HBSS and incubated at room temperature for de-esterification (30 min). Experiments in U2OS cells (Fig. S2*A* and *B*) were done using the genetically encoded calcium indicator, GCaMP6M. Fluorescence was imaged on an Olympus IX81 microscope, and fluorescence changes (λ_ex_ = 488 nm (λ_em_ = 513 ± 15-nm bandpass) were monitored using a Yogokawa spinning disk confocal (CSU-X-M1N) and an Andor iXon Ultra 888 EMCCD camera. Data were expressed as a ratio (*F*/*F*_0_) of fluorescence at any given time (*F*) relative to fluorescence prior to drug addition (*F*_0_).

### Electrophysiology

For whole-cell current recordings, HEK293 cells were transfected with a plasmid encoding GFP or co-transfected with plasmids encoding GFP and *Sm*.TRPM_PZQ_. One day later, cells were replated onto round 18-mm glass coverslips. After overnight incubation, coverslips were secured in a recording chamber over a Nikon Eclipse TE200 inverted microscope. Cells were continuously superfused (6 ml/min) with an extracellular buffer consisting of 140 mm NaCl, 5 mm KCl, 2 mm CaCl_2_, 1 mm MgCl_2_, 10 mm HEPES, and 10 mm glucose (pH 7.4, 310 ± 3 mosm at room temperature). HEK293 cells were held at a holding voltage of −40 mV, and responses were resolved after superfusion of extracellular buffer containing ±PZQ (2 μm). Recordings were made using borosilicate pipettes (Sutter Instrument Company, Novato, CA) pulled on a Sutter micropipette puller (model P-87) to resistances of 2–5 megaohms. Patch pipettes were filled with intracellular buffer containing 135 mm KCl, 10 mm NaCl, 1 mm MgCl_2_, 1 mm EGTA, 0.2 mm Na.GTP, 2.5 mm ATP.Na_2_, and 10 mm HEPES (pH 7.20, 290 ± 3 mosm at room temperature). Cell capacitance was compensated, and series resistance was kept <10 megaohms. Cells were included in analyses if the leak current stayed <200 pA. Recordings were made using an EPC10 USB amplifier (HEKA Electronics) and Patch Master software (HEKA Electronics). Patch-clamp data were analyzed using Pulse, PulseFit, or Fitmaster software (HEKA Electronics). For current-voltage measurements of HEK293 cells expressing *Sm*.TRPM_PZQ_, step potentials of 250 ms spanning the voltage range from −80 to +120 mV were delivered from a holding potential of −80 mV. For I-V curves, patch pipettes were filled with intracellular buffer containing: 140 mm CsMeSO_4_, 1 mm MgCl_2_, 1 mm EGTA, 10 mm HEPES-CsOH (pH 7.2 with CsOH, 300–310 mOsm/kg adjusted with sucrose).

## Author contributions

S. K. P. and J. S. M. conceptualization; S. K. P., G. S. G., E. G. C., F. M., and P. M. investigation; P. I. D. resources; J. D. C., C. L. S., and J. S. M. supervision; J. D. C., C. L. S., and J. S. M. writing-review and editing; J. S. M. funding acquisition; J. S. M. writing-original draft; J. S. M. project administration.

## Supplementary Material

Supporting Information

## Footnotes

2 The abbreviations used are:

PZQ: praziquantel
TRP: transient receptor potential
AITC: allyl isothiocyanate
TRPM: TRP melastatin
TM: transmembrane
ADPR: ADP-ribose
HEK: human embryonic kidney
HBSS: Hanks' balanced salt solution.
