# Supplementary material for: The anthelmintic drug praziquantel activates a schistosome transient receptor potential channel
Source: J Biol Chem. 2019 Oct 25;294(49):18873–80. doi: 10.1074/jbc.AC119.011093 (PMC6901322; doi:10.1074/jbc.AC119.011093)
Supplement: Supporting Information [file supp_294_49_18873__index.html]

The anthelmintic drug praziquantel activates a schistosome transient receptor potential channel — (R)-PZQ activates a schistosome TRP — The anthelmintic drug praziquantel activates a schistosome transient receptor potential channel — ACCELERATED COMMUNICATION: (R)-PZQ activates a schistosome TRP — Supporting Information 

# The anthelmintic drug praziquantel activates a schistosome transient receptor potential channel

## Supporting Information

- Supplementary Figure 1 - Supplementary Figure 1
- Supplementary Figure 2 - Supplementary Figure 2
- Supporting Information (to be published online) - Supplementary Figure Legends
