# Supplementary figures and images for: The anthelmintic drug praziquantel activates a schistosome transient receptor potential channel
Source: J Biol Chem. 2019 Oct 25;294(49):18873–80. doi: 10.1074/jbc.AC119.011093 (PMC6901322; doi:10.1074/jbc.AC119.011093)

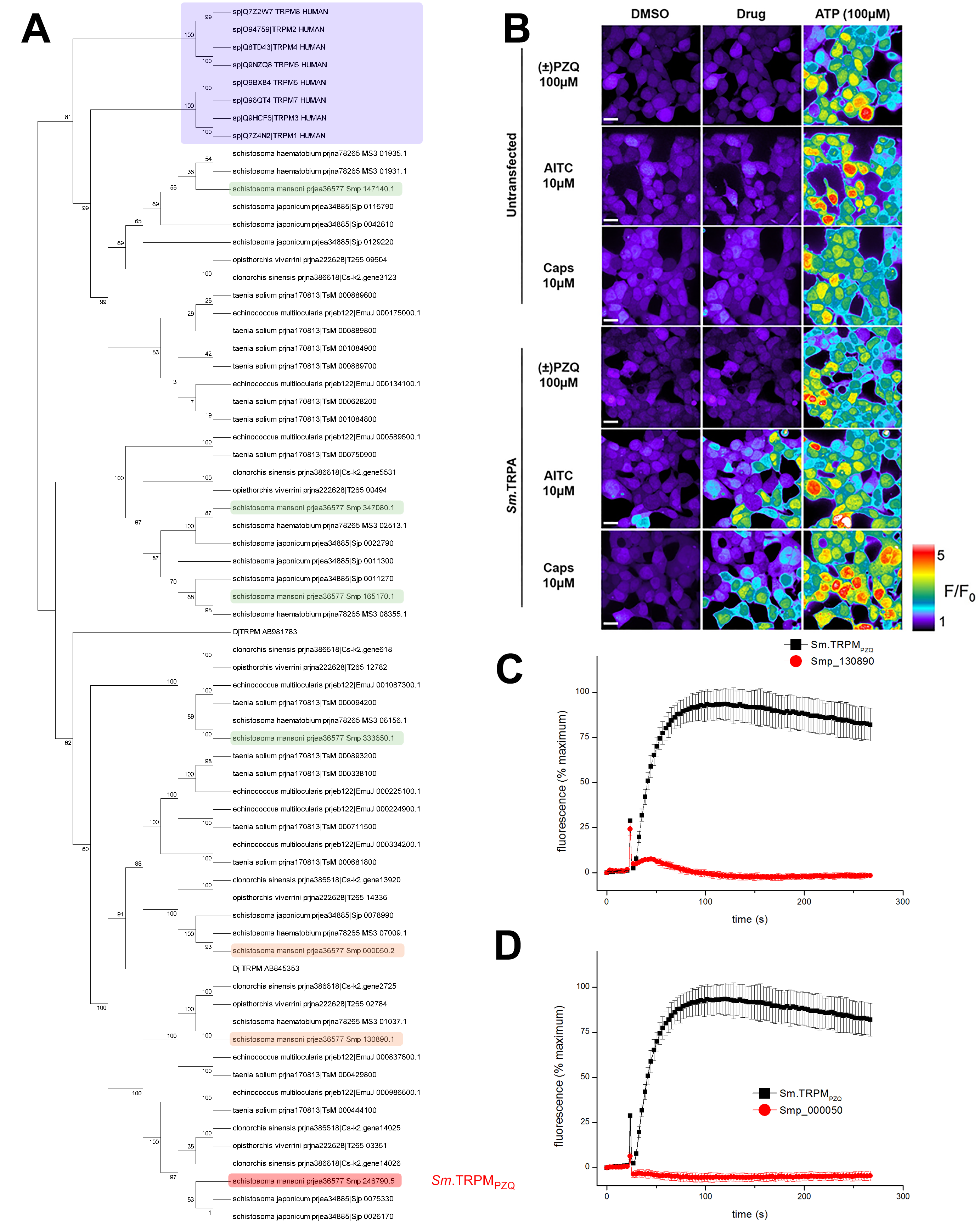

Supplement: Supporting Information [file supp_AC119.011093_155876_2_supp_417134_pzzz07.tif]

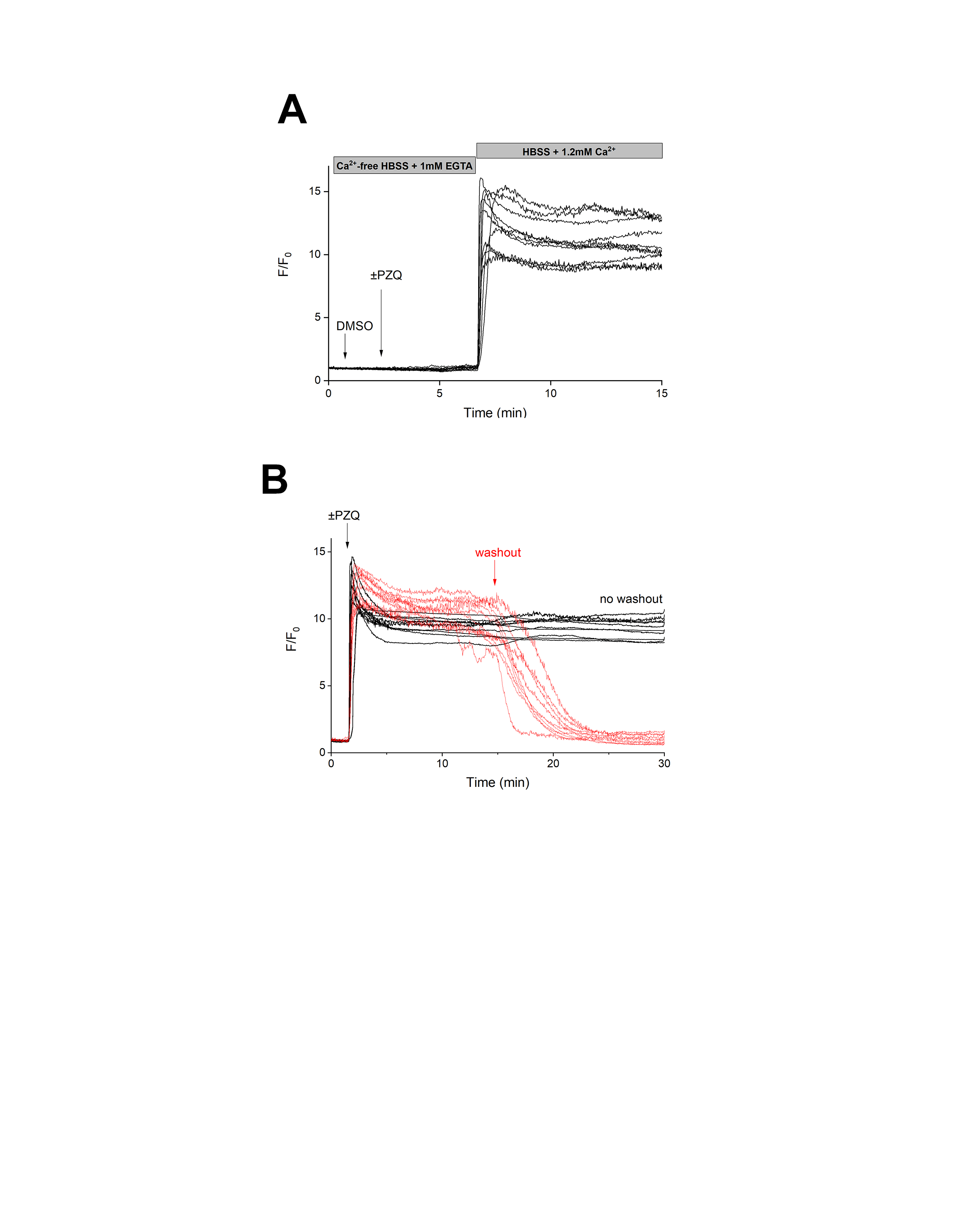

Supplement: Supporting Information [file supp_AC119.011093_155876_2_supp_417137_pz4409.tif]
