# Supplementary material for: The anthelmintic drug praziquantel activates a schistosome transient receptor potential channel
Source: J Biol Chem. 2019 Oct 25;294(49):18873–80. doi: 10.1074/jbc.AC119.011093 (PMC6901322; doi:10.1074/jbc.AC119.011093)

**Supplementary Data. Park *et al.***

**Supplementary Figure 1. Analysis of other flatworm TRP channels.** (**A**) *Sm*.TRPM_PZQ_ is present in other PZQ-sensitive flatworms. Rooted maximum-likelihood tree showing interrelationship between flatworm TRPM channels clustered with human TRPM channels, Sequences were obtained through BLAST searches against trematode (*C. sinensis*, *O. viverrini*, *S. mansoni*, *S. japonicum, S. haematobium*) and cestode (*E. multilocularis*, *T. solium*) genomes using human TRPMs. Results were ordered with duplicates removed prior to reciprocal BLASTs against human genome to filter out non-TRPM hits. TRPM sequences for *Dugesia japonica* were sourced from Inoue *et al* (35). Sequences were aligned using MUSCLE and degapped prior to tree construction with 500 bootstraps. Highlights indicate human TRPM channels (blue). Seven predicted *S. mansoni* TRPM channels are shown (green, orange and red). *Sm*.TRPM_PZQ_ is shown in red and other screened *Sm*.TRPM channels in orange (Supplementary Figure 1C&D). (**B**) PZQ does not activate *Sm.*TRPA. Pseudocolored confocal images of HEK293 cells loaded with fluo-4-AM. Fluorescence intensity was continuously monitored after addition of DMSO (0.05%) followed by addition of ±PZQ (100µM), or AITC (10µM) or capsaicin (10µM) and then ATP (100µM) in either untransfected HEK293 cells or cells transfected with plasmid encoding *Sm.*TRPA. Pseudocoloring indicates fluorescence ratios, F/F_0_, where ‘F’ represents fluo-4-AM fluorescence after addition of the indicated compound and ‘F_0_’ represents fluorescence at time = 0. Scale bar, 15µm. (**C&D)** PZQ does not activate two closely related TRPM channels. Fluo-4 fluorescence traces from cells expressing (C) Smp_138090 or (D) Smp_000050 monitored after addition of ±PZQ (100µM). Responses from *Sm*.TRPM_PZQ_ are shown from the same experiment for comparison. Results are averaged technical replicates from a single experiment, representative from three independent transfections.

**Supplementary Figure 2. Confocal imaging of single cell Ca^2+^ responses to PZQ treatment.** (**A**) *Sm*.TRPM_PZQ_ supports Ca^2+^ entry across the plasma membrane. U2OS cells, transiently co-expressing the genetic Ca^2+^ indicator GCaMP-6M and *Sm.*TRPM_PZQ_ , treated with PZQ (1µM) in Ca^2+^-free extracellular solution do not display a Ca^2+^ signal until extracellular Ca^2+^ is replenished. (**B**) Action of PZQ is reversible. Removal of PZQ (1µM) from the bathing medium decreased *Sm*.TRPM_PZQ_ evoked Ca^2+^ signals. Representative traces of GCaMP6M fluorescence from U2OS cells transiently expressing *Sm.*TRPM_PZQ_ treated with PZQ (1µM), either with (red) and without drug washout (black) beginning after 15min. Data are representative traces from multiple, single cells from an individual experiment selected from three independent transfections.


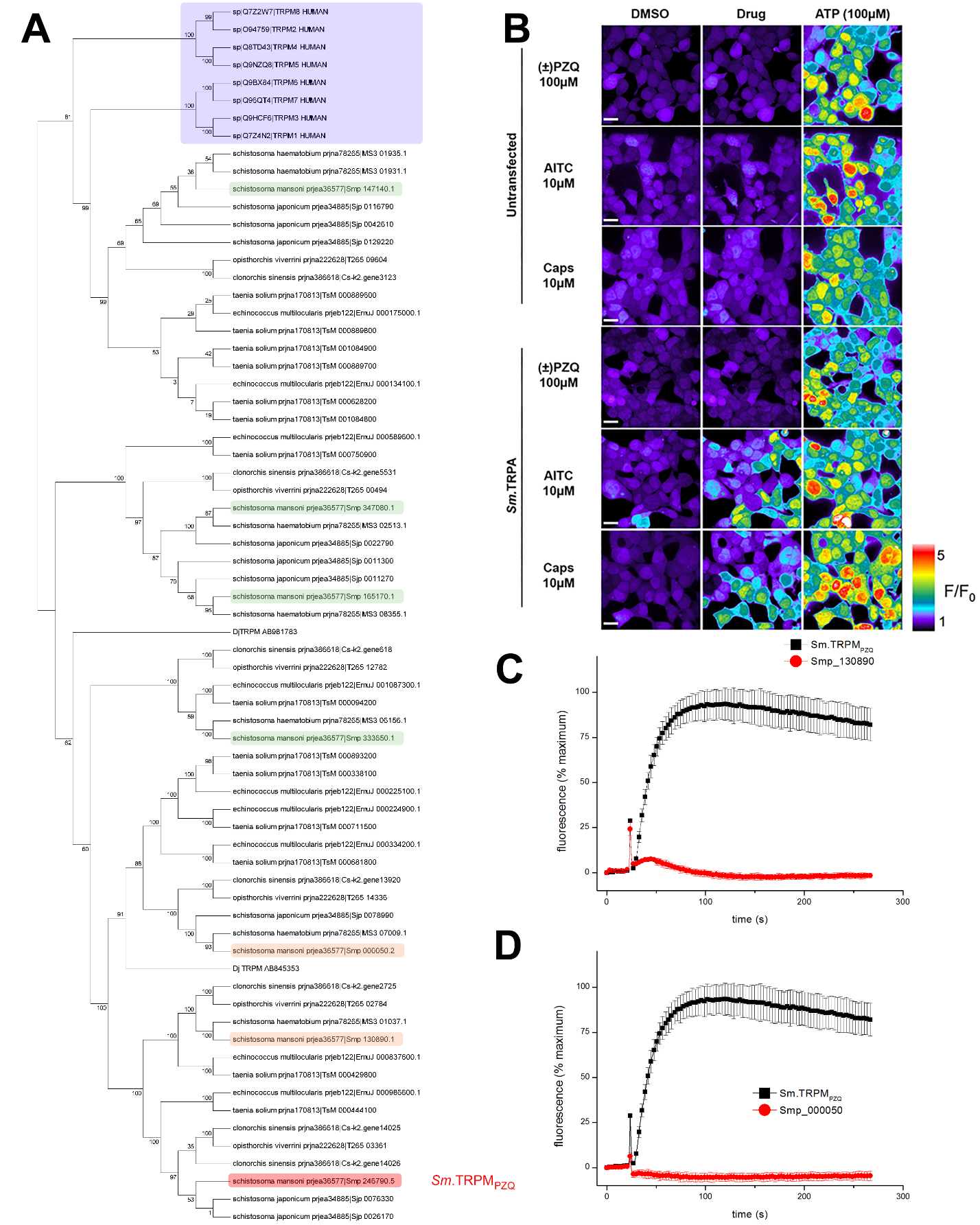


Supplementary Figure 2


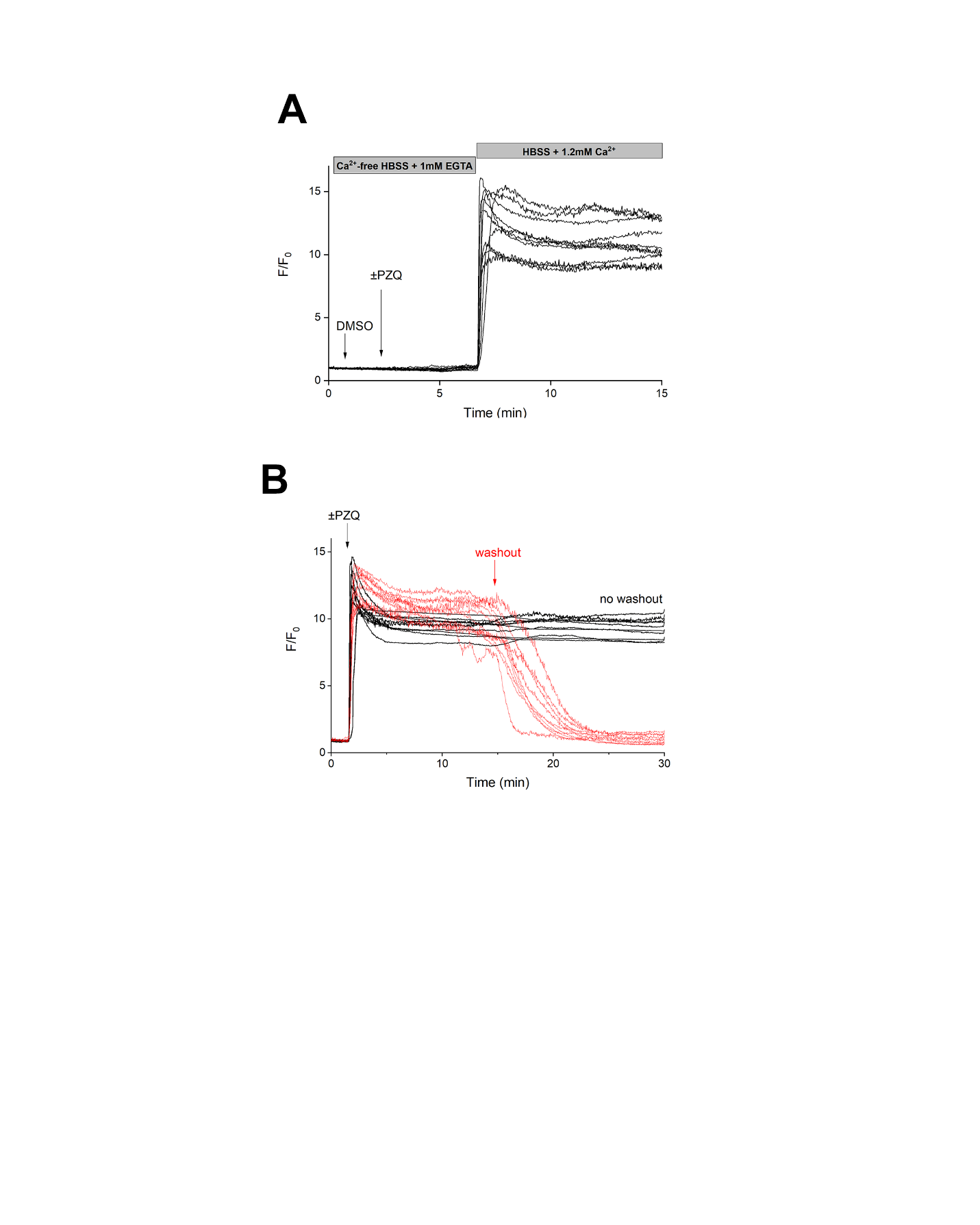

Supplement: Supporting Information [file supp_AC119.011093_155876_2_supp_417905_pzw7ff.docx]
